# CarE1 and GST1 Are Involved in Beta-Cypermethrin Resistance in Field Populations of the Mirid Bug, *Apolygus lucorum*

**DOI:** 10.3390/insects17010066

**Published:** 2026-01-06

**Authors:** Haojie Wang, Weicheng Song, Qiyuan Wu, Liming Xu, Lin Niu, Qingbo Tang

**Affiliations:** 1 Henan International Joint Laboratory of Green Pest Control, College of Plant Protection, Henan Agricultural University, Zhengzhou 450046, China; 2 State Key Laboratory of High-Efficiency Production of Wheat-Maize Double Cropping, Zhengzhou 450046, China

**Keywords:** *Apolygus lucorum*, beta-cypermethrin, detoxification metabolism, insecticide resistance

## Abstract

The widespread cultivation of transgenic Bt cotton has elevated *Apolygus lucorum* (Meyer-Dür) to a major pest in cotton agroecosystems. Its rapidly developing resistance to insecticides poses a serious challenge to sustainable agriculture. In this study, we assessed the susceptibility of a field-collected population from Anyang, Henan Province, in relation to a laboratory-susceptible strain, to elucidate the present status and molecular basis of resistance to beta-cypermethrin. First, the toxicity of beta-cypermethrin to *A. lucorum* was assessed through a diet-incorporation method. Subsequently, the enzymatic activities of carboxylesterase (CarE) and glutathione S-transferase (GST) were measured, and the expression levels of *CarE1* and *GST1* were quantified by quantitative real-time PCR (qRT-PCR). Finally, the function of candidate genes was confirmed using RNA interference (RNAi) technology. The bioassays results indicated that the median lethal concentration (LC_50_) for the laboratory and Anyang field strain were 343.34 mg/L and 700.45 mg/L, respectively. Following 48 h of exposure to the LC_30_ of the susceptible strain, the mortality rate of the field population (20.00%) was significantly lower than that of the laboratory population (33.33%), suggesting an increase in resistance. The field population of *A. lucorum* exhibited significantly higher activities of CarE (1.61-fold) and GST (1.71-fold) compared to the laboratory strain, accompanied by 3.63- and 4.23-fold overexpression of the corresponding genes *CarE1* and *GST1*. Spatiotemporal expression profiling revealed that *CarE1* expression was highest in 4th–5th instar nymphs and adults, with predominant localization in the midgut, while *GST1* expression peaked in 4th–5th instar nymphs and was abundant in the midgut and fat body. RNAi-mediated knockdown of *CarE1* and *GST1* significantly enhanced susceptibility to beta-cypermethrin in field populations, resulting in elevated mortality 48 h post-treatment compared to controls. In conclusion, the field population of *A. lucorum* has developed considerable resistance to beta-cypermethrin, strongly correlated with overexpression of *CarE1* and *GST1*. These results deepen our understanding of metabolic resistance mechanisms and offer valuable insights for developing targeted pest control strategies.

## Simple Summary

The mirid bug *Apolygus lucorum* has become an important pest in cotton cultivation, especially due to the widespread cultivation of Bt cotton. This species of insect bug has developed increasing resistant to insecticides, posing a growing threat to agriculture. In the present study, we investigated the mechanism underlying its resistance to beta-cypermethrin. We discovered that a field-collected population of *A. lucorum* survived insecticide exposure at significantly higher rates than the laboratory sensitive strain. The resistance is associated with increased expression levels of two detoxification genes, *CarE1* and *GST1*. Silencing these genes increased the susceptibility of pests to insecticides. Our results demonstrate that *CarE1* and *GST1* are key contributors to beta-cypermethrin resistance in *A. lucorum*, providing new insights for developing safer and effective pest control strategies.

## 1. Introduction

The polyphagous mirid bug, *Apolygus lucorum* (Meyer-Dür) (Hemiptera: Miridae), is a major agricultural pest widely distributed across eastern Asia. In China, it occurs predominantly in the Yangtze and Yellow River regions [1], where it causes substantial yield losses to a range of crops, including cotton. Known for its extensive distribution and high impact, *A. lucorum* represents a serious threat to agricultural production in China [2].

With the widespread cultivation of transgenic Bt cotton, the mirid bug *A. lucorum* has escalated from being secondary to primary pests in cotton fields, with their adverse effects extending beyond cotton to encompass various economically important crops, such as vegetables and fruit trees [2,3,4,5]. This shift in pest status has led to the strengthening of control strategies aimed specifically at mirid bugs, consequently increasing reliance on chemical insecticides in agricultural systems [6].

To combat this escalated threat, the increased reliance on chemical insecticides has led to the development of resistance in mirid bug populations to multiple classes of these compounds [7,8,9,10,11,12]. Specifically, populations of the green mirid bug, *A. lucorum*, have demonstrated significant regional resistance to pyrethroid insecticides. For instance, research conducted by Zhang Shuai et al. revealed that a population from Binzhou (Shandong) population exhibits moderate resistance to lambda-cyhalothrin, with a resistance ratio of 95-fold [13], whereas the Yancheng (Jiangsu) population displayed resistance levels ranging from 3.4- to 8.5-fold against beta-cypermethrin. Furthermore, resistance intensities were notably higher in the Binzhou (Shandong) and Cangzhou (Hebei) populations, with beta-cypermethrin resistance ratios between 7.1- and 22-fold, and chlorpyrifos resistance ratios ranging from 5.2- to 20-fold [12]. These results indicate that the ongoing escalation of insecticide resistance, combined with the lack of effective management interventions, presents considerable obstacles to the control of mirid bugs [14].

Insecticide resistance among agricultural pests represents a longstanding and critical challenge in the study of insect adaptation, posing significant threats to global food security [15]. Pests develop resistance to insecticides through various mechanisms, with target-site resistance and metabolic resistance-primarily driven by the upregulation of detoxification enzymes-being the predominant pathways [16,17,18]. The insect detoxification system largely involves cytochrome P450 monooxygenases (P450 s), carboxylesterases (CarEs/COEs), glutathione S-transferases (GSTs), UDP-glycosyltransferases (UGTs), and ATP-binding cassette (ABC) transporters [19,20,21,22,23]. Carboxylesterases play a crucial role in the metabolic detoxification of several classes of insecticides, including pyrethroids, organophosphates, and carbamates [24,25]. For example, in the Chinese chive root maggot, *Bradysia odoriphaga*, malathion exposure significantly upregulated the transcription of the *BoαE1* gene. Functional silencing of *BoαE1* increased the susceptibility of *B. odoriphaga* to malathion, and gas chromatography-mass spectrometry (GC-MS) analyses confirmed that BoαE1 possesses hydrolase activity against malathion [26]. Similarly, in the brown planthopper, *Nilaparvata lugens*, the *CarE17* gene was found to be overexpressed in resistant populations. RNA interference-mediated knockdown of *CarE17* enhanced susceptibility to chlorpyrifos, whereas heterologous expression of *CarE17* in transgenic *Drosophila* conferred increased tolerance to this insecticide [27]. Glutathione S-transferases also contribute substantially to metabolic resistance against diverse insecticides. For instance, exposure to lambda-cyhalothrin induced elevated expression of several *GST* genes-including *CpGSTd1*, *CpGSTd3*, and *CpGSTe3*-in the codling moth, *Cydia pomonella*. In vitro assays demonstrated that recombinant forms of these GST proteins are capable of metabolizing lambda-cyhalothrin [28]. In the oriental fruit fly, *Bactrocera dorsalis*, the *BdGSTd9* gene was overexpressed in a malathion-resistant strain; silencing of *BdGSTd9* increased susceptibility to malathion. Subsequent high-performance liquid chromatography (HPLC) analyses revealed that BdGSTd9 can degrade malathion and reduce levels of its metabolite, malaoxon [29]. Building upon this established knowledge of detoxification enzymes in other pests, we hypothesize that similar metabolic mechanisms underpin β-cypermethrin resistance in *A. lucorum*. Therefore, a systematic investigation into the specific roles of detoxification genes in this species is essential to elucidate the fundamental mechanisms driving its resistance evolution.

To investigate the resistance level and mechanisms of *A. lucorum* to beta-cypermethrin, we first evaluated the insecticide’s toxicity against both field-collected and laboratory populations using a diet-incorporation bioassay. Based on the bioassay results, we identified and screened differentially expressed genes potentially involved in detoxification through qRT-PCR analysis. Subsequently, candidate genes selected from this screening were then functionally characterized using RNA interference (RNAi)-mediated silencing. These findings enhance our understanding of the role of detoxification genes in the resistance of *A. lucorum* to beta-cypermethrin, and provide insights for developing more sustainable pest management strategies.

## 2. Materials and Methods

### 2.1. Insect

The laboratory strain of *A. lucorum* was initially established from adults collected from cotton fields at the Zhengzhou station of the Cotton Research Institute, Chinese Academy of Agricultural Sciences in 2020. This laboratory colony has been continuously maintained and purified for over 30 generations under laboratory conditions, without any exposure to chemical insecticides prior to this study. Insects were reared in climate-controlled incubators set at 26 ± 1 °C, 70% relative humidity, and a 15 h:9 h (light:dark) photoperiod, and provided with green beans and corn as dietary sources. A field population of *A. lucorum* was obtained from cotton fields in Anyang (Ay), China in late August 2023, and were reared under the same feeding conditions as the laboratory population for subsequent experiments. In Anyang, cotton fields are predominantly planted with transgenic Bt cotton, which has led to *A. lucorum* transitioning from a minor pest to a major one [5]. In recent years, the control of mirid bugs in these fields has primarily relied on the application of insecticides such as beta-cypermethrin, imidacloprid, methomyl and chlorpyrifos [30]. The first generation of third-instar nymphs derived from adults of the field population were used for subsequent biological assays.

### 2.2. Toxicity Assessment of Apolygus lucorum

Prior to bioassay, the first day of third-instar nymphs of laboratory and field colony of *A. lucorum* was acclimated at 25 °C for 24 h, provided with green beans as dietary sources. Toxicity tests were conducted using a treated diet method. Beta-cypermethrin (Shanghai Yuanye Biotechnology, Shanghai, China) was dissolved in acetone and serially diluted with an aqueous solution containing 0.1% Tween-80 (Solarbio Lifesciences, Beijing, China); a 0.1% Tween-80 solution served as the blank control. Fresh long beans were washed and immersed in the prepared insecticide solutions for 30 s to ensure uniform coating, followed by natural air drying. After the pre-adaptation period, treated beans were transferred to clean rearing containers housing the test insects. Each concentration was tested using thirty nymphs per replicate, with three replicates per concentration. Mortality was monitored and recorded at predetermined intervals (Nymphs of *A. lucorum* were considered dead if they showed no movement upon gentle touch with a brush and had their legs curled). The LC_50_ and LC_30_ values of beta-cypermethrin against both *A. lucorum* strains were determined at 48 h post-treatment across a concentration gradient (0, 30, 120, 480, 1920, and 7680 mg/L) using Probit method.

To further assess the resistance level of the field-derived *A. lucorum* populations from Anyang to beta-cypermethrin, a bioassay was performed using the LC_30_ concentration previously estimated for the laboratory population. The experiment was designed with three replicates, each containing 30 third-instar nymphs from the field population of *A. lucorum*. The laboratory population served as a control.

### 2.3. Determination of Detoxifying Enzyme Activity

#### 2.3.1. Enzyme Preparation

To investigate the detoxification-related enzyme activity in response to beta-cypermethrin exposure, fresh long beans treated with the LC_30_ concentration of the insecticide were provided as a diet to various populations of *A. lucorum* third-instar nymphs. After 48 h of exposure, ten nymphs from the laboratory or Anyang field strains were homogenized in 500 μL of 0.1 M ice-cold sodium phosphate buffer (pH 7.2) and protease inhibitor in a 2.0 mL centrifuge tube. The homogenates were then centrifuged at 10,000× *g* for 20 min at 4 °C, and the resulting supernatant was transferred to a 1.5 mL centrifuge tube. This supernatant was used as the enzyme source for the CarE and GST assay. Resulting supernatants were used for protein concentration determination at 595 nm (OD 595) using the Bradford assay kit (Tiangen, Beijing, China) with an Eon microplate reader (Biotek, Winooski, VT, USA).

#### 2.3.2. CarE and GST Enzyme Activity Assays

CarE activity was assayed with α-naphthyl acetate (α-NA) as the substrate, following a previously described procedure [31]. For the enzymatic reaction, 100 μL of the supernatant prepared from 2.3.1 was combined with 500 μL of 30 mM α-NA. Control wells received phosphate buffer instead of the enzyme solution. After incubation at 30 °C for 30 min in the dark, the reaction was terminated by adding 100 μL of a color-developing agent (a 5:2 mixture of 5% SDS and 1% fast blue B). The amount of α-naphthol (α-NP) produced was quantified by measuring the absorbance at 600 nm after 30 min, with reference to an α-NP standard curve. CarE activity was calculated and expressed as mmol of α-NP formed per mg of protein.

GST activity was measured using CDNB (1-chloro-2,4-dinitrobenzene) as the substrate according to a modified method based on Zhu et al. [32]. The reaction was performed in a total volume of 120 μL containing 10 μL of enzyme solution, 10 μL of 2 mmol L^−1^ CDNB, 50 μL of 10 mmol L^−1^ GSH, and 50 μL of 0.1 mol L^−1^ sodium phosphate buffer (pH 7.5). The absorbance at 340 nm was monitored at 10-s intervals over 10 min using a Synergy HTX microplate reader. Enzyme activity was calculated using an extinction coefficient of 5.3 mM^−1^ cm^−1^ for CDNB.

### 2.4. Detoxification-Related Genes Expression Measured by qRT-PCR

To investigate the detoxification-related gene expression in response to beta-cypermethrin exposure, fresh long beans treated with the LC_30_ concentration of the insecticide were provided as a diet to various populations of *A. lucorum*. After 48 h of exposure, the expression levels of the *CarE1* (GenBank No. PX758180) and *GST1* (GenBank No. PX758181) genes were quantified using quantitative real-time PCR (qRT-PCR). The qRT-PCR protocol consisted of an initial denaturation at 95 °C for 30 s, followed by 40 cycles of 95 °C for 10 s and 60 °C for 30 s. Each treatment group consisted of three biological replicates, with the *β-actin* gene and *RIBOSOMAL PROTEIN L32 (RPL32)* serving as the internal reference control [33,34]. Standard curves were prepared using a five-fold dilution series to determine the primer efficiencies (Supplementary Table S1).

To examine the expression patterns of the *CarE1* and *GST1* genes across various developmental stages and tissues in field population of *A. lucorum*, total RNA was isolated from different developmental phases (1st to 5th instar nymphs and adults) as well as from dissected tissues of 3rd-instar nymphs (head, thorax, abdomen, fat body, midgut, and legs) by using Trizol reagent (Takara Bio Inc., Otsu, Japan). The extracted RNA was reverse transcribed using PrimeScript^TM^ RT Master Mix (Takara Bio Inc., Otsu, Japan) to generate complementary DNA (cDNA). Expression levels of both genes were assessed using qRT-PCR method under the same cycling conditions as described above. Each treatment contained 3 replicates with 10 insects per replicate.

### 2.5. RNA Interference

To functionally validate the roles of *CarE1* and *GST1* in β-cypermethrin resistance, RNA interference was employed. Specific fragments of 439 bp of *CarE1* and 377 bp of *GST1* (primers in Table 1) were amplified using TaKaRa EX Taq polymerase (Takara Bio Inc., Otsu, Japan). The purified PCR products were subsequently used as templates for double-stranded RNA (dsRNA) synthesis following the protocol of the T7 RiboMAX^TM^ Express RNAi System (Promega Corporation, Madison, WI, USA).

Next, 0.4 ug of the synthesized dsRNA (2 μg/μL) in 0.2 ul was microinjected into third-instar nymphs of the field populations of *A. lucorum*. Control groups received the same amount injections of dsGFP or ddH_2_O (After microinjection, the survival rate of *A. lucorum* nymphs was higher than 85% after 48 h in all treatments, results see Supplementary Table S2). At 48 h post-injection, qRT-PCR analysis confirmed efficient silencing of the target genes (Gene knockdown efficiency for individual nymphs are provided in Supplementary Table S3. After injection of dsCarE1, 86.7% of nymphs showed interference efficiency exceeding 50%, while for dsGST1 injection, the proportion was 83.3%). After successfully reducing the expression levels of *CarE1* and *GST1* genes (≥50% reduction in expression), the 3rd nymphs of the *A. lucorum* were subjected to subsequent bioassays with a LC_30_ sublethal concentration of beta-cypermethrin. Mortality rates were recorded and analyzed by ANOVA to evaluate the differences between RNAi and control groups (each treatment contained 3 replicates with 30 nymphs per replicate), thereby elucidating the roles of *CarE1* and *GST1* in the insect’s response to the insecticide.

### 2.6. Data Analyses

Bioassay data and the determination of LC_50_ values were conducted utilizing SPSS version 26.0 software in accordance with Probit method. Spatiotemporal expression patterns were analyzed employing the 2^−ΔΔCt^ method as described by Schmittgen and Livak (2008) [35], and statistical significance was assessed through one-way analysis of variance (ANOVA) followed by Tukey’s honestly significant difference (HSD) post hoc test. Additionally, independent samples *t*-tests (comparison between two treatments) were applied to compare gene expression levels and the enzyme activities between experimental and control groups of *A. lucorum.*

## 3. Results

### 3.1. Detection of LC50 in Different Populations of Apolygus lucorum

The toxicity of beta-cypermethrin to *A. lucorum* was assessed using a diet-immersion bioassay over a 48 h exposure period (Table 2). The LC_50_ value for the laboratory strain was 343.34 mg/L, whereas the field-collected strain from Anyang showed an LC_50_ of 700.45 mg/L. These results indicate that the field population possessed a significantly higher level of resistance to beta-cypermethrin, with a resistance ratio (RR) of 2.04-fold compared to the laboratory strain (Table 2).

### 3.2. Detection of Resistance Level of Field Population

**Figure 1:**
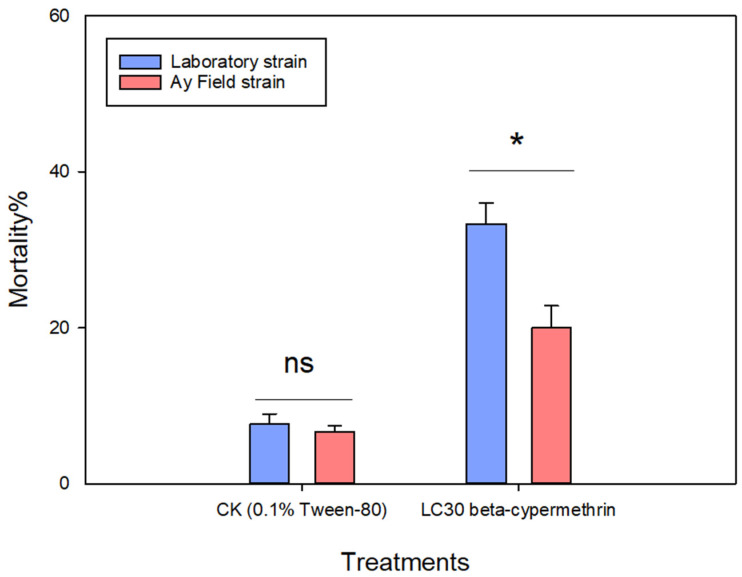
Mortality of *Apolygus lucorum* following 48-h exposure to LC_30_ treatment. Data are presented as mean ± SEM. Ns represents no significant difference (*p* > 0.05), * represents significant difference (*p* < 0.05), as determined by an independent samples *t*-tests. Values represent the means ± SEM from three replicates.

To further assess the resistance level of the field population of *A. lucorum* to beta-cypermethrin, both field-collected and laboratory-maintained populations were exposed to either a 0.1% Tween-80 aqueous solution (control) or the LC_30_ concentration of beta-cypermethrin (77.16 mg/L). The findings revealed that, after 48 h of exposure to the LC_30_ concentration, the mortality rate in the field population was 20.00%, which was significantly lower than the 33.33% observed in the laboratory population (Figure 1). These results suggest that the Anyang field population of *A. lucorum* has developed resistance to beta-cypermethrin.

### 3.3. Detection of CarE and GST Enzyme Activity

**Figure 2:**
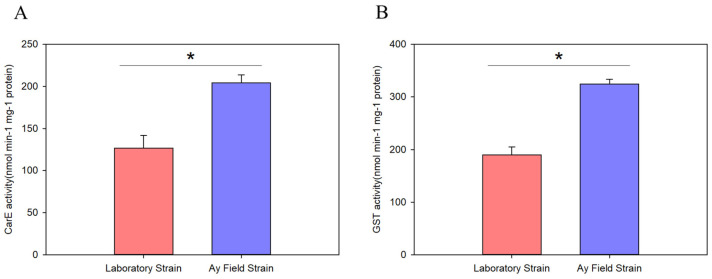
The enzyme activities of the CarE (**A**) and GST (**B**) were examined in different populations of *Apolygus lucorum*, including the laboratory strain and Anyang field strain. Data are expressed as mean ± SEM from three independent biological experiments. * represents a significant difference (Student’s *t*-test, * *p* < 0.05).

To investigate whether other CarE and GST enzymes contribute significantly to beta-cypermethrin resistance, we compared the activities of these enzymes between field and laboratory populations of *A. lucorum*. The results showed that CarE activity in the field population was 1.61-fold higher than that in the laboratory population (126.7 vs. 204.3 nmol/min/mg protein; Figure 2A). Similarly, GST activity was 1.71-fold greater in the field population compared to the laboratory population (190.0 vs. 324.3 nmol/min/mg protein; Figure 2B).

### 3.4. Detection of CarE1 and GST1 Gene Expression

**Figure 3:**
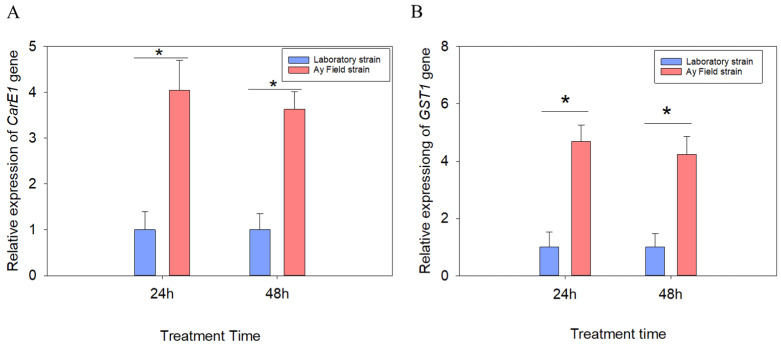
The expression levels of the *CarE1* (**A**) and *GST1* (**B**) genes following exposure to the LC_30_ concentration of beta-cypermethrin. Data are presented as mean ± SEM. Ns indicates no significant difference (*p* > 0.05) and * indicates a significant difference (*p* < 0.05), as determined by an independent samples *t*-tests. Each value represents the mean ± SEM derived from a minimum of three independent replicates.

To elucidate the potential role of *CarE1* and *GST1* in the reduced sensitivity of the field population of *A. lucorum* to beta-cypermethrin, their expression profiles were analyzed via quantitative real-time PCR (qRT-PCR) at 24 and 48 h after insecticide exposure. The findings revealed that the expression levels of both *CarE1* and *GST1* were significantly up-regulated in the field population compared to the laboratory population. Specifically, at 48 h post-treatment, *CarE1* expression was 3.63-fold higher (*p* < 0.05; Figure 3A), and *GST1* expression was 4.23-fold higher (*p* < 0.05; Figure 3B), in the field population relative to the laboratory controls.

### 3.5. Spatial and Temporal Expression Patterns of CarE1 and GST1 Genes in Apolygus lucorum

**Figure 4:**
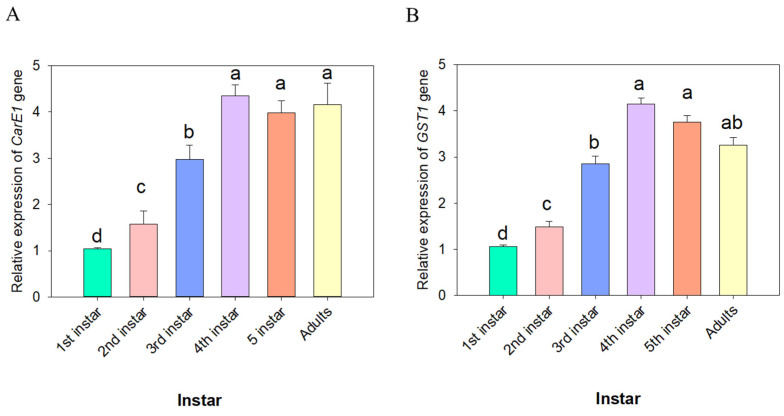
The expression levels of the *CarE1* (**A**) and *GST1* (**B**) genes across various instars of nymphs of *A. lucorum*. The data represent the mean relative expression of the *CarE1* and *GST1* genes ± standard error. Different letters above the bars denote statistically significant differences (*p* < 0.05), as determined by one-way analysis of variance followed by Tukey’s HSD post hoc test.

**Figure 5:**
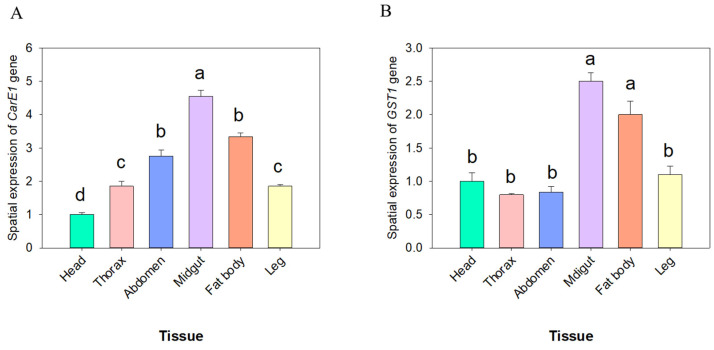
The expression levels of the *CarE1* (**A**) and *GST1* (**B**) genes across distinct tissues of third- instar nymphs of *A. lucorum*. The data represent the mean relative expression ± standard error. Different letters above the bars denote statistically significant differences (*p* < 0.05), as determined by one-way analysis of variance followed by Tukey’s HSD post hoc test.

The expression patterns of the *CarE1* and *GST1* genes across developmental stages and tissues were investigated using quantitative real-time PCR (qRT-PCR). The analysis revealed that *CarE1* gene expression was highest in the 4th–5th instar nymphs and adults, whereas it was lowest in the 1st-instar nymphs (Figure 4A). In contrast, *GST1* gene expression reached its highest level in the 4th, 5th-instar nymphs and was lowest in the 1st-instar nymph stages (Figure 4B). Further tissue-specific analysis in 3rd-instar nymphs revealed that *CarE1* expression was highest in the midgut and lowest in the head (Figure 5A). On the other hand, *GST1* expression was most prominent in the midgut and fatbody, with significantly lower levels detected in other tissues (Figure 5B).

### 3.6. Effects of RNA Interference on the Mortality of Apolygus lucorum

**Figure 6:**
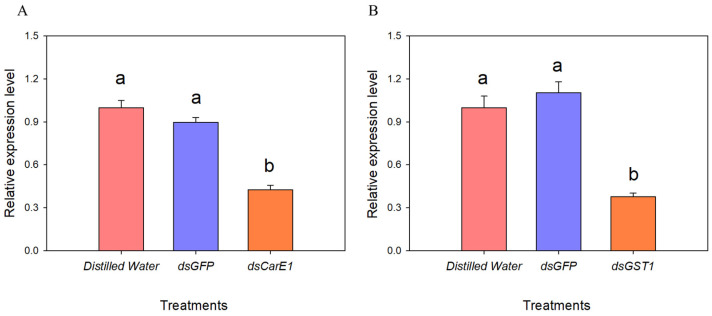
Detection of silencing efficiency of *CarE1* (**A**) and *GST1* (**B**) genes. The data represent the mean relative expression ± standard error. Different letters above the bars denote statistically significant differences (*p* < 0.05), as determined by one-way analysis of variance followed by Tukey’s HSD post hoc test.

To elucidate the molecular functions of *CarE1* and *GST1* in the metabolic detoxification of beta-cypermethrin, RNA interference (RNAi)-mediated silencing was employed in *A. lucorum*. After injection with dsCarE1 and dsGST1 for 48 h, the expression levels of the two genes were measured. The results showed that the expression of both genes was reduced by more than 50% (*CarE1* down by 58%, *GST1* down by 62%), indicating successful gene silencing (Figure 6).

**Figure 7:**
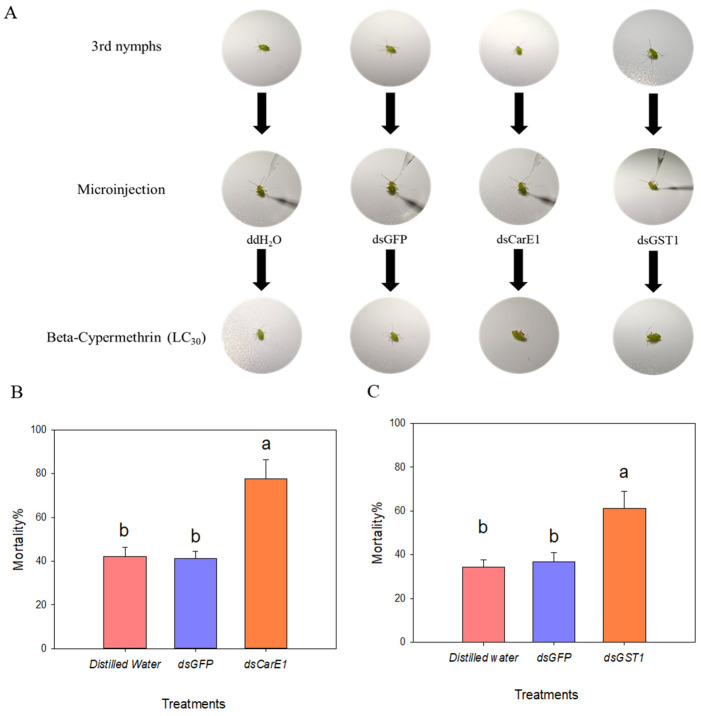
(**A**) illustrates the treatment of third-instar *Apolygus lucorum* nymphs with beta-cypermethrin at the LC_30_ concentration following dsRNA injection. (**B**) and (**C**) respectively shows the susceptibility to beta-cypermethrin of third-instar nymphs from the field population after knockdown of the *CarE1* and *GST1* genes. (**A**) shows that the 3rd-instar *A. lucorum* was injected with ddH_2_O, dsGFP and dsRNA, and then treated with beta cypermethrin at the concentration of LC_30_ to detect the mortality. A nymph was recorded as dead when it showed no movement after being gently touched with a brush and its legs remained curled together. Columns labeled with different letters in (**B**,**C**) denote statistically significant differences (*p* < 0.05), as determined by one-way analysis of variance followed by Tukey’s HSD test.

After confirming successful interference with both genes, nymphs of *A. lucorum* with *CarE1* or *GST1* silenced were separately exposed to an LC_30_ concentration of beta-cypermethrin. Mortality was assessed 48 h post-exposure. Silencing of *CarE1* in the field population resulted in approximately 40% higher mortality compared to the control (Figure 7B). Similarly, suppression of *GST1* also led to a significant increase in mortality (Figure 7C).

## 4. Discussion

Enhanced metabolic detoxification, mediated by enzymes such as glutathione S-transferases (GSTs) and carboxylesterases (CarEs), represents a common resistance mechanism in polyphagous insect pests following xenobiotic exposure [36]. Given the observed beta-cypermethrin resistance in the Anyang population of *Apolygus lucorum*, we investigated the potential involvement of these detoxification enzymes. Empirical evidence indicates that overexpression of *CarE* and *GST* genes is closely associated with insecticide resistance in various insect species [37,38]. Specifically, CarE-mediated resistance may involve enhanced hydrolytic activity, increased sequestration capacity, or altered enzyme-substrate affinity [39,40], with elevated *CarE* gene expression frequently correlating with resistance phenotypes [41,42]. Meanwhile, GSTs contribute to detoxification through glutathione conjugation, facilitating the production of less toxic, water-soluble metabolites. Additionally, GSTs mitigate oxidative stress by detoxifying reactive peroxides and may provide protection via non-catalytic insecticide binding [43].

The development of resistance to beta-cypermethrin in the *A. lucorum* field population from Anyang is likely mediated by the enhanced metabolic detoxification capabilities associated with the *CarE1* and *GST1*. Both Enzyme activity assay and qPCR analysis revealed significantly higher expression of both enzymes in the field-resistant population compared to the susceptible laboratory strain, supporting their role in the resistance phenotype. This correlation between gene overexpression and insecticide resistance is consistent with reports in other pests. For example, *CarE* gene expression was notably upregulated in abamectin-resistant strains of the terrestrial mollusk *Philomycus bilineatus* [44], while *GST* gene expression was substantially increased in the tarnished plant bug, *Lygus lineolaris*, following exposure to pyrethroid and neonicotinoid insecticides [45]. Such cross-species consistency suggests that upregulation of *CarE* and *GST* genes represents an evolutionarily conserved adaptive response to insecticide selection pressure. Therefore, the heightened expression of *CarE1* and *GST1* likely constitutes a key molecular mechanism conferring beta-cypermethrin resistance in the Anyang field population of *A. lucorum*.

The spatial expression patterns of *CarE1* and *GST1* further supports their functional importance in detoxification. The predominant localization of *CarE1* and *GST1* expression in the midgut—the primary site for digestion and initial insecticide exposure—aligns with findings in other insects, such as *Bombyx mori* [46]. This spatial configuration suggests that efficient detoxification occurs at the first point of toxicant entry, thereby reducing the effective insecticide concentration before systemic distribution.

Most importantly, RNA interference experiments provided direct causal evidence linking these genes to resistance. Silencing of *CarE1* or *GST1* significantly increased mortality (by 77–84%) in the field population upon beta-cypermethrin exposure, demonstrating their essential role in the detoxification process. This functional validation not only corroborates the correlation derived from expression profiling but also confirms the contribution of these enzymes in insecticide resistance.

Beyond the metabolic contributions of *CarE1* and *GST1* demonstrated here, resistance to pyrethroids like beta-cypermethrin in field populations of hemipteran pests often involves a complex interplay of multiple mechanisms. Target-site insensitivity, particularly knock-down resistance (kdr) mutations in the voltage-gated sodium channel, is a well-established cause of pyrethroid resistance and can coexist with enhanced metabolic detoxification [47]. Although not investigated in the present study, the potential presence of such mutations in the Anyang population cannot be ruled out and warrants future genotyping efforts. Furthermore, other major detoxification enzyme families, notably cytochrome P450 monooxygenases (P450s) [21] and UDP-glucuronosyltransferases (UGTs) [19], are frequently upregulated in resistant insects and may act independently or synergistically with CarEs and GSTs. The absence of synergist bioassays (e.g., using piperonyl butoxide or diethyl maleate) and direct in vitro metabolism data in our current work precludes a definitive assessment of their relative contributions and a direct biochemical confirmation of *CarE1*/*GST1*-mediated metabolism of beta-cypermethrin. These aspects represent important avenues for subsequent research.

Additionally, while our RNAi experiments (with ≥50% knockdown efficiency achieved in 83.3–86.7% of treated nymphs; see Supplementary Table S3) provide strong functional support, the reliance on a single geographical population necessitates caution in generalizing the findings. Regional differences in insecticide use history, ecological pressures, and genetic background may shape distinct resistance mechanisms in other *A. lucorum* populations. Therefore, expanding surveillance to include multiple field populations across different agricultural regions is crucial for developing comprehensive and regionally tailored resistance management strategies.

In conclusion, the constitutive overexpression of *CarE1* and *GST1*, their primary localization in the midgut, and the restored insecticide susceptibility following their suppression together indicate that metabolic detoxification is a key mechanism contributing to beta-cypermethrin resistance in *A. lucorum*. These findings provide new molecular insights into resistance development in *A. lucorum* and establish a foundation for designing targeted management strategies—such as those based on specific enzyme inhibitors or RNA interference. Incorporating such approaches into integrated pest management frameworks may offer sustainable solutions for controlling this pest.

## Figures and Tables

**Table 1:** Primer sequences used for qRT-PCR and RNA interference.

Gene	Orientation	Primer Sequences (5′–3′)	Application
*β-actin*	Forward	CGCCGACAGGATGCAGAAG	qRT-PCR
	Reverse	CGAGGATGGAGCCACCGA	qRT-PCR
*RPL32*	Forward	CAAGCTCACGAGGAATTGGCG	qRT-PCR
	Reverse	GAGGACTTTCCTGAAGCCGGT	qRT-PCR
*CarE1*	Forward	TCGCATCATCTACTCCGTTCACC	qRT-PCR
	Reverse	GTCCACGTTCATCAGAGAGGTCA	qRT-PCR
*GST1*	Forward	CTGTTCAGGACCTACACTCTCGC	qRT-PCR
	Reverse	TCACCGACATGTAAATGGTTATTCC	qRT-PCR
*CarE1*	Forward	gcgtaatacgactcactatagggGGATTGGACGGCATACAACC	RNAi
	Reverse	gcgtaatacgactcactatagggGTTGCCTTTGTACTGCTCCC	RNAi
*GST1*	Forward	gcgtaatacgactcactatagggACTCATGAATGTTATCTCGCTGG	RNAi
	Reverse	gcgtaatacgactcactatagggTAGCGGGTTGGGGAACGAC	RNAi

**Table 2:** Toxicity of beta-cypermethrin against Laboratory and Anyang field Strains of *Apolygus lucorum* after 48 h of treatment.

Strain	Toxicity Regression Equation	LC50 (mg/L) (95% CI)	RR	X^2^ Value	*p* Value
Laboratory Strain	y = 0.002x − 0.680	343.34 (267.24–433.52)	1.0	528.47	<0.01
Ay field Strain	y = 0.001x − 0.771	700.45 (492.53–976.94)	2.04	125.25	<0.01

Lethal concentration to achieve 50% mortality (LC_50_) values and 95% confidence intervals were calculated by Probit analyses using SPSS software. Resistance ratio (RR) calculated by dividing LC_50_ of the Anyang field strain by LC_50_ of Laboratory strain.

## Data Availability

The original contributions presented in this study are included in the article/Supplementary Material. Further inquiries can be directed to the corresponding authors.

## Supplementary Materials

The following supporting information can be downloaded at: https://www.mdpi.com/article/10.3390/insects17010066/s1, Table S1. Specifications for optimized qRT-PCR amplification of *A. lucorum* genes. Table S2. The survival rate of the nymph of *A. lucorum* after injection of ddH_2_O, dsGFP, dsCarE1 or dsGST1 for 48 h. Table S3. Gene knockdown efficiency (per individual) in *Apolygus lucorum* nymphs after injection of *dsCarE1* or *dsGFP1*.
