# Supplementary material for: CarE1 and GST1 Are Involved in Beta-Cypermethrin Resistance in Field Populations of the Mirid Bug, Apolygus lucorum
Source: Insects. 2026 Jan 6;17(1):66. doi: 10.3390/insects17010066 (PMC12841984; doi:10.3390/insects17010066)
Supplement: Supplementary file 1 [file insects-17-00066-s001.zip › insects-4044229-supplementary.pdf]

Supplementary Data

This Supplementary Information contains:

Supplementary Tables S1-S3

Supplementary Table S1. Specifications for optimized qRT-PCR amplification of *A. lucorum* genes.

Gene name	Efficiency	R2	Slope
β-actin	106.9%	0.999	-3.168
RPL32	92.9%	0.999	-3.506
CarE1	99.9%	0.995	-3.324
GST1	94.6%	0.996	-3.459

Supplementary Table S2. The survival rate of the nymph of *A. lucorum* after injection of ddH_2O, dsGFP, dsCarE1 or dsGST1 for 48 h.

Treatment	Total number of insects injected	Number of surviving insects	Survival rate
ddH_2O	120	105	87.5%
dsGFP	120	108	90%
dsCarE1	120	112	93.3%
dsGST1	120	110	91.7%

Supplementary Table S3. Gene knockdown efficiency (per individual) in *Apolygus lucorum* nymphs after injection of *dsCarE1* or *dsGFP1*.

Types of injected dsRNA	Serial number of *A. lucorum* nymphs	Gene knockdown efficiency (%)	Percentage of nymphs exhibiting gene knockdown efficiency exceeding 50% (%)
dsCarE1	1	65.4	86.7
	2	61.4	
	3	74.8	
	4	61.4	
	5	74.3	
	6	64.3	
	7	74.4	
	8	55.7	
	9	61.7	
	10	67.1	
	11	23.2	
	12	75.6	
	13	65.1	

	14	43.5	
	15	16.3	
	16	56.3	
	17	61.1	
	18	61.4	
	19	64.0	
	20	62.9	
	21	61.9	
	22	63.1	
	23	66.5	
	24	57.0	
	25	70.8	
	26	67.2	
	27	66.7	
	28	45.8	
	29	62.5	
	30	32.5	

Types of injected dsRNA	Serial number of *A. lucorum* nymphs	Gene knockdown efficiency (%)	Percentage of nymphs exhibiting gene knockdown efficiency exceeding 50% (%)
dsGST1	1	73.6	83.3
	2	87.1	
	3	84.5	
	4	64.0	
	5	64.5	
	6	64.3	
	7	21.1	
	8	61.5	
	9	62.8	
	10	63.7	
	11	75.4	
	12	86.9	
	13	89.8	
	14	39.3	
	15	74.3	
	16	79.9	
	17	82.0	
	18	84.1	
	19	32.1	
	20	64.8	
	21	65.4	
	22	77.9	
	23	64.1	

24	27.9
25	61.2
26	62.5
27	62.0
28	26.0
29	74.8
30	81.6
